# The New Anæsthetic

**Published:** 1880-05

**Authors:** W. E. Riggs

**Affiliations:** Atlanta, Ga.


### THE NEW ANÆSTHETIC.

By W. E. Riggs, D.D.S., Atlanta, Ga.

(Read before the Georgia Dental Society, May 13, 1880.)

Ether, chloroform and nitrous oxide gas have been used with great good resulting, but they are all more or less dangerous and exceedingly inconvenient at times. It is not necessery to go into details on this head, as those who have used either or all, know such is the fact.

My object in addressing you is to speak of the superiority—I say superiority, for in my opinion it far excels any anæsthetic I have used—of bromide of ethyl.

Bromide of ethyl, or hydrobromic ether, was discovered by Serullas in 1827 and introduced to the profession by Dr. Nunnely, of Leeds, Eng., in 1849. This ether is a light volatile liquid, made by distilling four parts of powdered bromide of potassium with five parts of a mixture consisting of two parts of strong sulph. acid and one of alcohol, having a boiling-point of 105° Fah., it is nearly involible in the blood. This ether is of interest from the fact that the late Dr. Nunnely, of Leeds, Eng., proposed and used it as a general anæsthetic, and came to the conclusion that it was the best and safest of all known anæsthetic substances.

Dr. B. W. Richardson says that he had the pleasure of visiting Dr. Nunnely, a few weeks before his death and in the course of many conversations on scientific subjects, he spoke again of his experience with the bromide of ethyl, and begged Dr. Richardson to submit it to a fair and strict investigation.

Dr. Richardson carried out his wishes, and reports that it is, as Dr. Nunnely said of it, one of the safest of general anæsthetics. An atmosphere containing from eight to nine per cent. of the vapor of the bromide of ethyl causes, when inhaled, entire destruction of common sensibility rapidly and safely. The breathing remains tranquil, the pulse quiet, the expression good; the transition from the first to the third degree of narcotism is, moreover, so rapid that the second degree—degree of muscular excitement—is scarcely recognizable.

As might be expected, from the low boiling point of the ether, 105° Fah., and its involibility in the blood, it is rapidly eliminated from the body when it has been withdrawn, so that the period of recovery is short—from two to five minutes.

Dr. Turnbull says "it is an anæsthetic which, with care, may be administered to man and animals. It is more rapid in producing anæsthesia than even chloroform, and is eliminated by respiration and the kidneys more rapidly than any other of this class of agents." The heart and respiration are but very slightly affected unless employed in excessive quantities.

Vomiting is more rare than with ether or chloroform.

The hydrobromic ether not being inflammable, and producing its anæsthetic influence on the muscles of the throat, any operation can be performed on the mouth and throat with satisfaction to the surgeon and comfort to the patient.

In one hundred cases of administration by Dr. Turnbull, there were twelve cases of slight nausea after the operation and eight cases of vomiting during the operation; but always where the patient had partaken freely of food of a solid character just prior to the use of the anæsthetic.

*Asphyxia.*—The disagreeable and painful symptom was not noticed in any of the cases.

*Fainting.*—In no instance was there any evidence of it.

*Hysterical Excitement.*—This was noticed in some six cases, but soon passed away, leaving no bad symptom.

The rapidity with which the patients came under the anæsthetic influence of the hydrobromic ether: ten came in one minute and a half, twenty in two minutes, ten in two and a half minutes, forty in three minutes, ten in four minutes, ten in five minutes.

He also states that in fifty cases, to recover consciousness, it took from two to two and a half minutes; in thirty cases, three minutes, and in twenty cases, four and one half minutes.

Struggling, coughing or gagging, which occurs so often during etherization, was very rare under the anæsthetic influence of hydrobromic ether.

This anæsthetic is not apt to produce headache; no giddiness attends the use of it.

Having had considerable experience in the administration of the various anæsthetics in use at the present day, viz., chloroform, sulphuric ether, nitruos oxide gas—and not feeling satisfied with the safety, or rather unsafety, of chloroform or with the many faults of sulphuric ether, which so nearly counterbalance its comparative safety as to preclude its use in favor of chloroform in many cases—I have, therefore, experimented with the hydrobromic ether, and am fully satisfied that it is the best anæsthetic known to the profession.

